# An Atlas of Bacteriology

**Published:** 1899-09

**Authors:** 


**An Atlas of Bacteriology.** By Chas. Slater, M.B., and Edmund J. Spitta. Pp. xiv., 120. London: The Scientific Press, Limited. 1898.

The value of this excellent book, which consists, in addition to its pages of letterpress, of a collection of one hundred and eleven full-page original photo-micrographs, is much enhanced by the brief introductory description of the method by which the pictures have been produced, a method which is in complete accord with the published experience of some of the most accomplished operators in this field of work. The saving of time, labour, and money resulting from the immediate possession of a knowledge of the best available means of obtaining satisfactory representations of the bacteria will render the acquisition of this volume a very profitable transaction on the part of anyone entering upon this fascinating study, although some acquaintance with the manipulation of microscope and camera will be required to put into practice the terse directions of the authors.

The perfection of the methods adopted is best attested by the beauty of the pictures, the truthfulness of which makes them very valuable companions to the text-book. A word of commendation is also due to the short descriptions of the organisms which accompany the illustrations, and which, without being overloaded with detail, furnish a fairly complete account of the leading features of the various species.

